# Oral Presentations 3

**DOI:** 10.1038/sj.bjc.6601929

**Published:** 2004-06-22


### ORAL PRESENTATIONS 3

### 3.1

### GENETIC ANALYSIS OF THE TYLOSIS WITH OESOPHAGEAL CANCER *(TOC)* GENE REGION ON 17Q25

F.E. McRonald [1], L. Rowbottom [1], J.E. Langan [1], A. Ellis [2], J.M. Shaw [2], J.K. Field [1], J.M. Risk [1]
[1] The University of Liverpool, Liverpool, United Kingdom,
[2] Royal Liverpool and Broadgreen University Hospital, Liverpool, United Kingdom

Tylosis (focal non-epidermolytic palmoplantar keratoderma) is associated with the early onset of squamous cell oesophageal cancer in two large families from the UK and US and a smaller German pedigree. The familial cancer association is rare, but the gene is also implicated in the development of sporadic squamous cell oesophageal cancer. Our recent haplotype and linkage analyses using 9 novel microsatellite markers and 52 novel single nucleotide polymorphisms have reduced the minimal region on chromosome 17q25 from ~500kb to ~38kb. This region contains one complete gene and partial coding sequences of two other genes. Mutational analysis of the coding regions of the candidate genes did not identify any disease-associated mutations. Sequencing of the 38kb region in UK and US family members has identified 2 genetic alterations for further investigation as potentially disease-causing, although neither of these is located within known gene coding regions. The gene that is contained entirely within the minimal region has recently been identified as cytoglobin (*CYGB*), or Stellate-cell Activation-associated Protein (*STAP*). Using RT-PCR, we have shown that *CYGB* is ubiquitously expressed, suggesting that its function is not merely a hepatic one. However, expression of *CYGB* is down-regulated in oesophageal biopsies from tylosis patients in comparison with normal oesophagus and is alternatively spliced. Although no disease-specific sequence changes are evident in the coding region of *STAP*, the regulatory and intronic regions are candidate sites for causative mutations.
The identification and characterisation of the *TOC* gene could, therefore, contribute to the understanding of this commonly occurring cancer. A molecular marker such as this could potentially have applications in cancer screening or rational drug design.

### 3.2

### ROLE OF GASTRIN IN *H. PYLORI* INDUCED HB-EGF GENE UP-REGULATION AND ECTODOMAIN SHEDDING

J Dickson [1], A Grabowska [1], J Atherton [2], SA Watson [1]
[1] Academic Unit of Cancer Studies, University of Nottingham, Nottingham, United Kingdom, [2] Division of Gastroenterology, University of Nottingham, Nottingham, United Kingdom

Heparin-binding epidermal growth factor-like growth factor (HB-EGF) is expressed in a propeptide form that is tethered to the cell membrane. The mature growth factor is cleaved to exert an effect on cells via binding to the EGF-receptor. This process can be induced by a number of factors including exposure to *H. pylori*. The aim of this work was to investigate the role of gastrin in *H. pylori* induced HB-EGF gene up-regulation and ectodomain shedding in gastric cells.
Wild-type gastric adenocarcinoma cell lines, AGS cells transfected to express human $CCK_2$ receptors (classical and Intron 4 isoforms) and gastric cells transfected with a gastrin anti-sense plasmid were co-cultured with either gastrin and/or the *cag*$^+$ *vacA* s1/m1 toxigenic strain 60190 or the *cag*$^-$ *vacA* s2/m2 nontoxigenic strain Tx30a of *H. pylori*. Western blotting and ELISA were used to determine levels of HB-EGF ectodomain shedding, whilst gastrin and HB-EGF gene expression were measured using real-time PCR. Statistical analysis was performed using one-way ANOVA.
*H. pylori* significantly up-regulated expression of HB-EGF and gastrin genes in wild-type gastric cells (p<0.003). Addition of exogenous gastrin with *H. pylori* further increased HB-EGF gene expression in cell lines that did not respond to gastrin alone (p<0.002). Analysis by Western blot and ELISA confirmed that this trend was repeated at the protein level (p<0.001).
AGS $CCK_2$ receptor transfected cells exhibited greater up-regulation of HB-EGF and gastrin gene expression than both the Intron 4 isoform and vector control transfected cell lines (p<0.004). Gastrin anti-sense cells displayed a lower degree of HB-EGF gene up-regulation and ectodomain shedding than vector control (p<0.001).
*H. pylori* can up-regulate gastrin and HB-EGF gene expression, and HB-EGF ectodomain shedding. This effect appears to be mediated by exogenous and cell-associated gastrin possibly via the CCK-2 receptor.

### 3.3

### THE EFFECT OF TRANSCATHETER ARTERIAL CHEMOEMBOLIZATION ON ANGIOGENESIS AND THE EXPRESSION OF VEGF IN HEPATOCELLULAR CARCINOMA

B Wang [1], ZQ Gao [2], H Xu [1], GW Cao [1], YQ Sun [1], DX Yu [1], HF Ning [1]
[1] Department of Radiology, the Affiliated Hospital, Weifang Medical University, Weifang, China, [2] Department of Medical Biology, Weifang Medical University, Weifang, China

**Introduction** Hepatocellular carcinoma (HCC) is one of the most common cancers in China and the Asian. The effection of the treatment in HCC both TACE and the surgical resection has some limitation. The objective of this study is to investigate intratumor microvessel density (MVD), expression of vascular endothelial growth factor (VEGF) in survival cancerous tissue in HCC after TACE.
**Methods** The specimens of HCC in 63 cases verified by histopathology were studied, which included 42 cases treated with surgical resection alone (the control group), and 21 cases undergoing 1-2 times TACE therapy prior to the surgical resection (the TACE group). The medicine and the emblization materials including 5-Fu, DDP, PHT-ADM, HCPT, lipiodol and absorbable gelatin sponge were infused through hepatic artery once time in the TACE group. The MVD, diameter of microvessel and VEGF protein expression in HCC tissue were measured respectively.
**Results** The MVD was 51.69 ± 18.17 in the control group and 58.57 ± 15.75 in the TACE group. There was no significant difference between the two groups ($t$ = 1.48, $P$ > 0.05). The diameter of microvessel was 17.62 ± 10.54 µm in the control group and 15.79 ± 7.65µm in the TACE group. There was no significant difference between the two groups ($t$ = 0.71, $P$ > 0.05). The cell number of VEGF expression was 243.66 ± 88.88 in the TACE group, that was higher than in the control group (138.26 ± 65.24) ($t$ = 5.34, $P$ < 0.01). There was a positive correlation between the expression of VEGF and MVD ($r$ = 0.4936, $t$ = 4.4329, $P$ < 0.05).
**Conclusion** The study indicates that the survival cancerous tissue in HCC after TACE has rich vascularity and expression of VEGF of the survival cancerous cells can be enhanced by TACE which may play an important role in re-establishment of blood supply to HCC after TACE.

### 3.4

### MICROARRAYS IDENTIFY PROGNOSTIC PROFILES IN COLON CANCER PATIENTS AND POTENTIAL MECHANISMS OF 5FU CHEMORESISTANCE

ESR Collie-Duguid [1], GI Murray [1], DS Stewart [1], D Bissett [2], J Cassidy [3], LM Samuel [2]
[1] University of Aberdeen, Aberdeen, United Kingdom, [2] Aberdeen Royal Infirmary, Aberdeen, United Kingdom, [3] University of Glasgow, Glasgow, United Kingdom

Chemoresistance to current 5-fluorouracil (5FU) based therapies is a significant clinical problem in colorectal cancer treatment, with only around 40% of patients alive 5 years after diagnosis. Informative and accurate pre-therapy data is required to guide clinical decisions and allow individualisation of patient treatment.
Expression levels of over 22000 transcripts were measured in surgical resection specimens of moderately differentiated tumour (T) and uninvolved adjacent (N) tissues from Dukes' C colon cancer patients, using Affymetrix HG-U133A GeneChips™. All patients received adjuvant 5-fluorouracil therapy following potentially curative resection of their primary tumour and were classified according to disease-free survival (long >45m or short <8m). Data was analysed using Affymetrix MASv5, MicroDBv3, DMTv3 and GeneSpringv6.1.
The expression patterns of a threshold and probability filtered subset of 17 genes differentiated tumour and uninvolved tissues in all patients in unsupervised hierarchical clustering and predicted histological diagnosis class with 100% accuracy and high predictive strength (p ratio <0.032), in leave one out cross-validation. A subset of genes, differentially expressed between long and short survivors in tumour tissues (≥ 2fold, p<0.01), differentiated patients with different survival phenotypes, using unsupervised hierarchical clustering. An accurate (100%) and strong (p ratio <0.12) prediction rule for patient survival was built from the expression patterns of these genes.
Supervised analysis of ontological groups important in tumorigenesis and/or 5FU chemoresistance, demonstrated that key factors, including cell cycle, apoptotic and pyrimidine metabolic genes, were associated with tumorigenesis or patient survival in colon cancer patients. Global examination of abnormal gene expression profiles in colon tumours, has identified potential molecular determinates of prognosis and suggested possible mechanisms of 5FU chemoresistance.

© 2004 Cancer Research UK

### 3.5

**QUASAR: A RANDOMISED STUDY OF ADJUVANT CHEMOTHERAPY (CT) VS OBSERVATION INCLUDING 3239 COLORECTAL CANCER PATIENTS**

The QUASAR Collaborative Group
United Kingdom, QUASAR Study Office, University of Birmingham, Birmingham

**Background:** It is unclear whether adjuvant fluorouracil/folinic acid (FUFA) CT produces worthwhile survival benefits for patients with node negative colorectal cancer. **Methods:** Patients with apparently curative resections of colon or rectal cancer were randomised following surgery between FUFA and observation (with CT considered on recurrence). CT consisted of either six 5-day, four-weekly or 30 once-weekly courses of intravenous fluorouracil (370mg/$m^2$) with either high-dose (175mg) or low-dose (25mg) L-folinic acid, and with either levamisole or placebo. Primary outcome was all cause mortality. **Results:** Between June 1994 and December 2003, 3239 patients (91% Dukes B, 71% colon cancer, median age 63) from 150 centres in 17 countries were randomised between CT and observation and 4927 between CT regimens. No benefit was seen for high-dose FA or levamisole (2000, *Lancet*; 355:1588). With a median follow-up of 4.2 years, risk of death with CT vs control was 0.85 (95%CI 0.72-1.00; p=0.05) and recurrence 0.81 (0.69-0.95; p=0.01). Treatment efficacy did not differ significantly by stage, site, age or schedule. Differences between CT and observation were seen only for QoL measurements directly related to toxicity (diarrhoea, nausea, vomiting, fatigue, appetite loss and mouth pain; all p<0.001), and only during CT. **Conclusions:** These inexpensive and relatively low toxicity chemotherapy regimens produce a small (1% - 5%) survival benefit for stage B patients, which is sufficient to outweigh the inconvenience and cost for high-risk and younger patients. Longer follow-up and meta-analysis of all trials is needed to clarify the balance of benefits and disbenefits for older patients.

### 3.6

**CAN MOLECULAR MARKERS PREDICT BOTH RESPONSE TO TREATMENT AND CLINICAL OUTCOME IN SQUAMOUS CELL CARCINOMA OF THE ANUS**

S Mawdsley [1], R Glynne-Jones [1], S Bentzen [1], F Daley [1], G Wilson [1], A Makris [1], H Meadows [3], R James [2]
[1] The Gray Cancer Institute, Middlesex, United Kingdom, [2] Anal Cancer Trials Management Group, London, United Kingdom, [3] CRUK, MRC & UCL trials centres, London, United Kingdom

**Aim**: The prognostic and predictive value of a panel of molecular markers and clinicopathological variables in anal cancer were explored.
**Methods**: Tumour samples from 230 patients entered into the UKCCCR ACT I trial were studied. 124 received radiation alone (RT) and 106 concurrent mitomycin/5FU/RT (CMT). T stages: T1:33, T2:80, T3:88, T4:29. Median age 63 yrs, male: female ratio 43:57%, median follow-up 46 months. An immunohistochemical analysis of p53, bcl-2, Ki67, thymidylate synthase, thymidine phosphorylase (TP), cyclins D1/A, DPD, CA9 and CD34 was undertaken. The primary endpoint was disease-free survival (DFS), with secondary endpoints of cause-specific survival (CSS) and treatment response.
**Results**: Several markers on multivariate analyses were independent predictors of each survival endpoint, see table. CD34 (p=0.014), T stage (p<0.001) and TP expression (p<0.001) predicted an improved DFS in the CMT arm. Stage (p<0.001) and p53 expression (p=0.030) predicted a poorer response to treatment and in the RT alone arm decreasing cyclin A (p=0.018) and increasing bcl-2 (p=0.030) also predicted for a poorer response. Response predicted survival (p<0.000).
**Conclusion**: This is one of the largest studies examining the role of molecular markers in anal cancer, demonstrating that outcome and treatment response can be predicted independently of standard clinicopathological characteristics. Making tailoring the treatment to the individual a future possibility.

| | DFS | P | HR | | CSS | P | HR | |
|---|---|---|---|---|---|---|---|---|
| | T | 0.000 | 1.77 | | T | 0.000 | 2.04 | |
| | N | 0.008 | 1.70 | | N | 0.008 | 1.74 | |
| | CD34 | 0.038 | 0.99 | | P53 | 0.021 | 1.28 | |
| | TP | 0.000 | 1.46 | | Tarm | 0.000 | 2.34 | |
| | Tarm | 0.000 | 2.8 | | | | | |

### 3.7

**DOES HIGHLY ACTIVE ANTIRETROVIRAL THERAPY (HAART) AFFECT THE INCIDENCE OR OUTCOME OF INVASIVE ANAL CANCER?**

A Waterston , C Thirlwell , T Newsom-Davis , T Powles , M Nelson , B Gazzard , M Bower
Chelsea & Westminster Hospital, London, United Kingdom

**Background:** The incidence of invasive anal carcinoma is increased amongst people with HIV. The effects of HAART on anal cancer incidence and outcome are unknown.
**Methods:** The anal cancer incidence in our prospective cohort of 8,640 HIV positive individuals (40,126 patient years of follow-up) was measured in pre (1984-95) and post (1996-2003) HAART eras and compared to the age and gender matched general population of SE England for 1995 (3.1 x$10^6$) collected by the Thames Cancer Registry.
**Results:** The incidence of anal cancer in our HIV+ cohort is 60/$10^5$ patient years (PY). This is 120 times higher than in the matched population. The incidence was 35 (95% CI: 15-72) /$10^5$ PY in the pre HAART era and 92 (95% CI: 52-149) /$10^5$ PY in the post HAART era: giving relative risks of 67 and 176 respectively (p<0.001 for both). 26 HIV+ patients with histologically confirmed invasive anal cancer were identified. The median age at presentation of anal cancer is 43 years (range 28-56) and CD4 cell count is 206 cells/$mm^3$ (16-749). 12 were receiving HAART but only 5 (42%) had undetectable HIV-1 viral loads. 22 patients were treated with chemoradiotherapy, 2 tumours confined to the anal verge were completely excised and 2 patients received palliative care only. Median follow-up is 4.8 years, 11 patients have died (7 from anal cancer and 4 in remission). The overall survival at 5 years is 47% (95% CI: 24-70%). There is no difference in overall survival between the pre and post HAART eras (Log rank *p*=0.19).
**Conclusions:** HAART has not reduced the incidence of anal cancer. This cohort study is the largest series reported thus far. The 5 year overall survival is worse than for the general population, however, the 2 year survival rate is 74% with no relapses of anal cancer occurring after this time and all subsequent deaths attributed to HIV infection. These results are encouraging as they include 2 patients who received palliative care only.

© 2004 Cancer Research UK

